# Mediating immunosuppressive functions: a new perspective on the complex immunological properties of SEMA4D in the tumor microenvironment

**DOI:** 10.3389/fonc.2023.1171926

**Published:** 2023-05-23

**Authors:** Shujie Zeng, Zihao Zhang, Chunshui Ye, Jinshen Wang, Changqing Jing, Leping Li

**Affiliations:** ^1^ Department of Gastrointestinal Surgery, Shandong Provincial Hospital, Cheeloo College of Medicine, Shandong University, Jinan, Shandong, China; ^2^ Department of Gastroenterological Surgery, Shandong Provincial Hospital Affiliated to Shandong First Medical University, Jinan, Shandong, China; ^3^ Key Laboratory of Engineering of Shandong Province, Shandong Provincial Hospital, Jinan, Shandong, China; ^4^ Medical Science and Technology Innovation Center, Shandong First Medical University & Shandong Academy of Medical Sciences, Jinan, Shandong, China

**Keywords:** SEMA4D, pancancer, tumor microenvironment, immune infiltration, exhaustion of CD8+ T cell

## Abstract

Semaphorin 4D (SEMA4D) is considered a new antitumor target closely related to immune cells. However, understanding the role of SEMA4D in the tumor microenvironment (TME) is limited. In this study, we explored the expression and immune cell infiltration patterns of SEMA4D using multiple bioinformatics datasets and analyzed the relationship between SEMA4D expression with immune checkpoints, tumor mutational load (TMB), microsatellite instability (MSI) and immune function. We detected that SEMA4D is overexpressed in many tumors types, widely enriched in immune cells, and closely associated with TILs, MSI, TMB, as well as T-cell exhaustion-associated immune checkpoints, and thus can broadly affect the immune microenvironment. We further verified the overexpression of SEMA4D in tumor and its distribution in TME by immunohistochemistry, RT-qPCR and flow cytometry, and confirmed that decreased expression of SEMA4D can lead to recovery of T cell exhaustion. In conlusion, this study provides a more comprehensive perspective of SEMA4D regulation of tumor immunity, which provide a new option for cancer immunotherapy.

## Introduction

Semaphorin 4D (SEMA4D), also called CD100, is a member of the semaphorin superfamily IV subfamily (1). SEMA4D has been confirmed to be expressed at high levels in a variety of tumors, including prostate cancer, colon cancer, breast cancer, oral cancer, lung cancer, pancreatic cancer, head and neck carcinoma, and soft tissue sarcoma (2). SEMA4D regulates the invasion and migration of tumor cells and participates in tumor angiogenesis through various mechanisms. It is a transmembrane semaphorin and has a 120 kDa soluble form (1). In addition, SEMA4D is not a redundant biomolecule in the immune system, and it is expressed at high levels in several autoimmune diseases and infections, including neuroinflammatory demyelination, systemic sclerosis, heart failure, haemorrhagic fever with renal syndrome, hepatitis C virus infection, multiple sclerosis, rheumatoid arthritis and Huntington disease (3).

SEMA4D was the first semaphorin reported to play a key role in the immune response (4). SEMA4D regulates immune cell function mainly through its receptors plexin and CD72. Most of its immunomodulatory effects are mediated by the CD72 receptor, which is expressed in B cells, dendritic cells (DCs) and monocytes, and it regulates humorall and cellular immunity by activating B cells and DCs (5). In addition, SEMA4D regulates immune cell migration through multiple mechanisms. Plexin receptors inhibit lymphocyte migration, while chemokines and membrane SEMA4D on lymphocytes stimulate lymphocyte migration (6, 7). SEMA4D also functions as a receptor, transmitting activation signals into lymphocytes and increasing T cell proliferation (8). SEMA4D widely mediates the suppressive tumor immune microenvironment.

Tumor cells and immune cells have complex interactions in the immune microenvironment, and SEMA4D expressed on tumor cells participates in editing the inhibitory immune microenvironment. Tumor cells secrete SEMA4D, which stimulates myeloid-derived suppressor cell (MDSC) differentiation, enhances the immunosuppressive function of MDSCs, and inhibits T cell proliferation and IFN-γ production. Blocking SEMA4D improves inhibitory immune microenvironments and produces a durable antitumor response (3). Therefore, SEMA4D plays a complicated role in the tumor microenvironment (TME) because it affects a wide range of immune cell populations and participates in stimulatory and inhibitory immune responses. Understanding the biological characteristics of the TME of different tumors will greatly facilitate predictions of the prognosis and guide treatment.

Here, we analyzed the expression patterns of *SEMA4D* in the TME and its relationship with immune cell infiltration, microsatellite instability (MSI), the tumor mutational burden (TMB) and immune checkpoints using bioinformatics methods combined with next-generation sequencing (NGS) data and single-cell sequencing across multiple cancers. Real time fluorescent quantitative polymerase chain reaction (RT–qPCR), immunohistochemical (IHC) and flow cytometry (FCM) were used to further confirm the expression pattern of SEMA4D in TME and to explore the effect of SEMA4D in tumor-infiltrating lymphocytes.

Real time fluorescent quantitative polymerase chain reaction (RT–qPCR), immunohistochemical (IHC) and flow cytometry (FCM) were used to further confirm the expression pattern of SEMA4D in TME and to explore the effect of SEMA4D in tumor-infiltrating lymphocytes.

## Materials and methods

### Data processing and differential expression analysis

Images of IHC staining for SEMA4D protein expression in 13 pairs of normal and tumor tissues, including normal liver and liver cancer, normal endometrium and endometrial cancer, normal colon and colon cancer, normal breast and breast cancer, normal skin and skin melanoma, normal lung and lung cancer, normal kidney and renal cancer, normal testicle and testicular cancer, normal ovary and ovarian cancer, normal lymph node and lymphoma, normal head-neck salivary gland and head-neck cancer, normal pancreas and pancreatic cancer, and caudate and glioma, were downloaded from the Human Protein Atlas (HPA) (http://www.proteinatlas.org/) and analysed. The antibody used for IHC of all sections was Anti-SEMA4D Antibody (Sigma-Aldirich, HPA015662). The protein expression score is based on IHC data manually scored for staining intensity (negative, weak, moderate or strong) and the fraction of stained cells (<25%, 25-75% or >75%) (9). The images on the atlas are the raw data that show the IHC. The expression score describes a knowledge-based best estimate of true protein expression, meaning that an expert has manually analyzed all images and assessed whether or not the staining is likely to represent true protein expression. To provide an overview of protein expression patterns, all images of tissues are manually annotated by one expert and then verified by a second expert. The annotation of each different normal and cancer tissue is performed according to fixed guidelines for classification of IHC results.

*SEMA4D* and checkpoint expression data as well as clinical information from cancer patients were collected from The Cancer Genome Atlas (TCGA) database.

### Relationship between SEMA4D expression and immunity

The correlation between immune and stromal scores was measured with the ESTIMATE algorithm using R software, The immune score and stromal score are calculated by an algorithm called ESTIMATE (Estimation of STromal and Immune cells in MAlignant Tumor tissues using Expression data) which is first proposed by Kosuke Yoshihara et al. in 2013 (10). We used CIBERSORT to analyze the relationship between *SEMA4D* expression and immune cell infiltration with TIMER2.0 (http://timer.cistrome.org/), and the data were visualized by constructing a heatmap and performing cluster analyses using Python.

The expression levels of *SEMA4D* in specific immune cells were investigated using the Tumor Immune Single-Cell Hub (TISCH) (http://tisch.comp-genomics.org/), a large-scale curated database that integrates single-cell transcriptomic profiles of nearly 2 million cells from 76 high-quality tumor datasets across 27 cancer types. Tumor datasets were mainly collected from Gene Expression Omnibus and ArrayExpress. All the data were uniformly processed with a standardized workflow (11). Uniform Manifold Approximation and Projection (UMAP) and heatmap were used to show *SEMA4D* expression in different immune cell types among tumors, and the data were visualized by constructing a heatmap and performing cluster analyses using Python.

### Correlation of SEMA4D expression with TMB and MSI

TMB was defined as the total number of coding errors in somatic genes, base substitutions, insertions or deletions detected per million bases (12). If the TMB is higher, the cancer cell contains more mutations, and immune cells more easily recognize and kill it. The TMB appears to be an important independent biomarker within MSI-H tumors that has been used to stratify patients based on the likelihood of a response to immune checkpoint inhibitors. MSI is also a biomarker of the response to immune checkpoint inhibitors (13). MSI scores were determined for all samples based on somatic mutation data downloaded from TCGA (https://tcga.xenahubs.net), and the relationships between *SEMA4D* expression and TMB and MSI were analyzed by calculating Spearman’s rank correlation coefficients.

### Clinical data and tissue sample

Clinical data and samples from 28 patients who had undergone radical (R0) resection with a histological diagnosis of colon adenocarcinoma (COAD) at the Shandong Provincial Hospital from July 2020 to September 2020 were retrospectively collected. The use of patient information and tissues was sanctioned by the Medical Ethics Committee of Shandong Provincial Hospital. The samples were used for IHC staining and RT–qPCR.

### Cell lines culture and lentivirus transfection

B16 is a C57BL/6 mouse melanoma cell line, and MC38 is a C57BL/6 mouse colon cancer cell line. These cell lines were provided by the laboratory of Shandong Provincial Hospital Center. B16 and MC38 cells were cultured at 37°C in an incubator with 5% CO_2_ in DMEM-H supplemented with 10% foetal bovine serum, 100 units/mL penicillin and 100 µg/mL streptomycin. The cells were routinely tested to confirm the absence of mycoplasma contamination and were cultured for a limited number of generations.

Knockdown lentivirus (sh-SEMA4D) and the negative control (sh-NC) were purchased from Genomeditech Inc. (Shanghai, China), Before infection, B16 cells were seeded in 6-well plates overnight, and then 1 ml fresh medium containing lentivirus (MOI:50-100) was added to each well. After 72 h, the successfully transfected cells were then screened with 2 μg/mL puromycin. Western blot and qRT-PCR were adopted to evaluate the transfection efficiency. All target site sequence, primers sequence and antibody were listed in **Supplementary Table 1**. The verification results were shown in **Supplementary Figure 2**.

### Mice and *in vivo* experiment

Six- to eight-week-old female C57BL/6 mice were supplied by Charles River (Beijing China) and raised in the Animal Center of Shandong Provincial Hospital. All animal experiments were approved by the Committee for Ethics of Animal Experiments of Shandong Provincial Hospital and complied with China’s current regulations and standards for the use of laboratory animals.

MC38 and B16 cells (1 × 10^6^) were collected within limited passages and then injected into the right back of mice in 100 µL of PBS(n=6~9). Tumor growth was observed after 5 days. Mice were euthanized after 14 days, and the tumors were collected for subsequent experiments.

B16 cells transfected with *SEMA4D* knockdown or control lentivirus subcutaneously injected into the right back of C57 mice (n=5). Tumor volumes were measured every other day starting after 5 days and the mice were euthanized after 14 days.

### RT–qPCR

Total RNA was isolated with Total RNA Extraction Reagent (Vazyme, China). Total RNA was reverse transcribed into cDNAs with a reverse transcription kit (Vazyme, China) and ChamQ Universal SYBR qPCR Master Mix for qPCR (Vazyme, China). The RT–qPCR primer sequences were as follows: *β-actin*, forward primer: 5’-CATGTACGTTGCTATCCAGGC-3’; reverse primer: 5’-CTCCTTAATGTCACGCACGAT-3’; and *SEMA4D* forward primer: 5’- GGAGCTCTGCACAAAGCCATC-3’, reverse primer: 5’-GCCCGAGTTAGAGCCAGCATAG-3’.

### Flow cytometry

Samples were obtained from blood, spleen, or tumors, as indicated. Tumor samples were weighed after harvest and digested/dissociated, and total cells were counted. Excess surface marker antibodies were mixed with cells and incubated at 4°C for 30 min. After treatment with the Cyto-Fast™ Fix/Perm Buffer Set (Biolegend 426803) or Foxp3/Transcription Factor Staining Buffer Set (Invitrogen 00-5523-00), intracellular markers or Nucleoprotein were stained with the corresponding antibodies. The panel included the following reagents: Zombie NIR™ Fixable Viability Kit, anti-CD45 (Biolegend 103128), anti-CD3 (Biolegend 100203) anti-CD4 (Biolegend 100539), anti-CD8a (Biolegend 100711), anti-CD100 (BD 745346), IgG2a,κ Isotype Control (BD 563236), anti-CD11B (Biolegend 101255), anti-F4/80 (Biolegend 123135), anti-CD11C (Biolegend 117317), anti-CD86 (Biolegend 105037), and anti-CD206 (Biolegend 141706), anti-PD-1 (Biolegend 135227), anti-LAG-3 (Biolegend 125209), anti-TIM-3 (Biolegend 119717), anti-TIGIT (Biolegend 142111).

### IHC

IHC assays were performed using paraffin-embedded COAD and matched normal tissues. An IHC kit (Zsgb Bio, Beijing, China) was used for IHC staining. Briefly, sections were stained with 3,3-diaminobenzidine tetrahydrochloride (DAB) and haematoxylin reagent after sequential incubations with primary (Abcam, ab134128, China) and secondary antibodies. The amount and intensity of staining in three fields of view were evaluated using a microscope (Olympus, Tokyo, Japan) at 200× magnification to score the IHC staining. Each field of view was scored for staining intensity and percentage of positive cells, and the overall score was the product of the staining intensity score and cell positivity score (staining intensity score: 0 for no staining, 1 for pale yellow staining, 2 for pale brown staining, and 3 for brown staining; cell positivity score: 0 to 5% was assigned 0 points, 6% to 25% was assigned 1 point, 26% to 50% was assigned 2 points, 51% to 75% was assigned 3 points, and >75% was assigned 4 points).

### Statistical analysis

The log_2_ transformation was used to normalize all gene expression data. Two sets of t tests were employed to compare normal and malignant tissues. Differences between multiple groups were assessed using one-way analysis of variance (ANOVA) (normal data) or the Kruskal–Wallis test (nonnormal distribution). Spearman’s or Pearson’s test was used to examine the correlation between two variables. Statistical significance was defined as p < 0.05. All statistical analyses were performed using R software (version 3.6.3) and GraphPad Prism 9.0.0 software.

Spearman’s correlation analysis was used to assess the correlation between SEMA4D expression and immune checkpoint genes. R > 0.20 was considered positively correlated, and p < 0.05 was considered statistically significant (*: p value < 0.05, **: p value <0.01, and ***: p value <0.001).

## Results

### Tissue expression of SEMA4D across cancers

**Figure 1:**
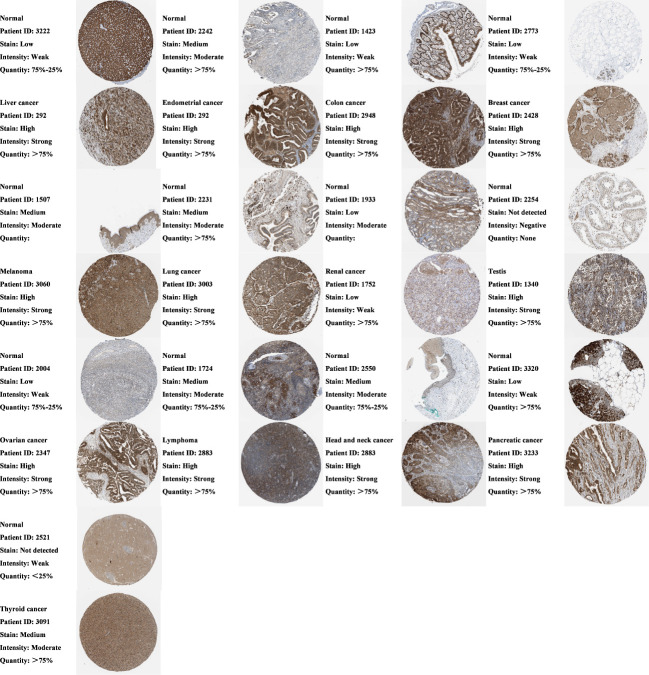
Protein expression of SEMA4D is increased in cancer tissues. Comparison of SEMA4D protein expression between normal and tumor tissues across cancers using IHC in Human Protein Atlas (HPA). The image on the right shows the raw IHC staining data, and on the left is the result of the expert scoring the image based on what was mentioned in the part of methods. Representative patterns are shown above, and the remaining replicates are shown in the **Supplementary Materials** (n=3).

Lu et al. analyzed the expression of *SEMA4D* mRNA in normal and tumor tissues (2). To further verify the results, we assessed the expression of SEMA4D at the protein level in normal and cancerous tissues using the HPA database. Normal and tumor tissues were stained with the same antibody for IHC, and differential expression of SEMA4D was analyzed to ensure accurate results. As shown in **Figure 1**, our IHC results demonstrated that SEMA4D was expressed at significantly higher levels in multiple cancer types, including liver cancer, endometrial cancer, colon cancer, breast cancer, skin melanoma, lung cancer, renal cancer, testicular cancer, ovarian cancer, lymphoma, head-neck squamous cell carcinoma, pancreatic cancer, and glioma, compared to the respective normal tissues (n=3).

We further analyzed the expression levels of *SEMA4D* in early and advanced stages of various tumors to investigate its potential role in tumor progression. Analysis of TCGA data revealed that *SEMA4D* was not expressed at higher levels in late stages compared to early stages in all tumor types examined. However, *SEMA4D* expression was significantly higher in early-stage lung adenocarcinoma (LUAD), skin cutaneous melanoma (SKCM) and testicular germ cell tumors (TGCT) tissues than in late stages, as shown in **Figure S1**.

### Primary source of SEMA4D in the TME

**Figure 2:**
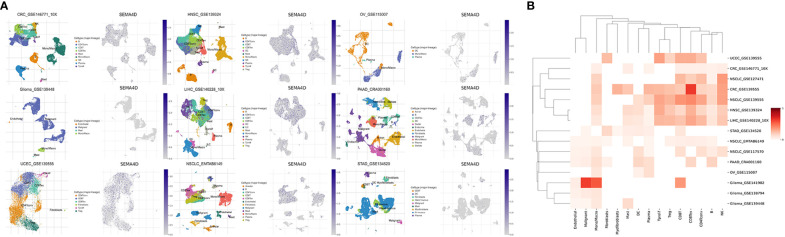
*SEMA4D* in tumors is mainly derived from TME. The distribution in the TME of tumors with high expression of *SEMA4D* was shown as above. **(A)** Two UMAP map with cells colored by cell type (left side) and *SEMA4D* expression level (right side) are displayed for representative datasets. **(B)** The heatmap shows *SEMA4D* expression in different cell types across all eligible datasets. Darker red indicates higher expression of *SEMA4D* in these immune cells in this dataset. The selected datasets were CRC_GSE139555, CRC_GSE146771_10X, Glioma_GSE138794, Glioma_GSE139448, Glioma_GSE141982, HNSC_GSE139324, LIHC_GSE140228_10X, NSCLC_EMTAB6149, NSCLC_GSE117570, NSCLC_GSE127471, NSCLC_GSE139555, OV_GSE115007_SEMA4D, PAAD_CRA001160, STAD_GSE134520 and UCEC_GSE139555).

To identify the primary source of *SEMA4D* in the tumor microenvironment (TME), we conducted a single-cell RNA sequencing (scRNA-seq) transcriptome analysis of 15 independent datasets of 9 tumors with high *SEMA4D* expression. We excluded non-compliant databases (immunotherapy or chemoradiotherapy, other species and inconsistent sequencing methods) and used TISCH to mine the scRNA-seq data. We then reduced the dimension of single cell and cell type data and used a two-dimensional UMAP map to show the expression of SEMA4D in different cell types in the TME. **Figure 2A** shows representative data sets for different tumor types. Different cell types were marked with different colors on the left side of the image, and the expression of *SEMA4D* corresponding to different cell types was shown on the right side. The heatmap in **Figure 2B** shows the expression of *SEMA4D* in different cell types across multiple datasets.

Our analysis revealed that *SEMA4D* was commonly expressed in immune cells. Specifically, it was most widely expressed in macrophages in multiple cancer types, such as colorectal cancer (CRC), non-small cell lung cancer (NSCLC), head and neck squamous cell carcinoma (HNSC), liver hepatocellular carcinoma (LIHC), pancreatic adenocarcinoma (PAAD), ovarian cancer (OV), and glioma. Moreover, SEMA4D was significantly enriched in T cells of various tumors, including CD8+ T cells, CD4+ conventional T cells (conv), and proliferating T cells (Tprolif). SEMA4D was also highly expressed in regulatory T cells (Tregs) and exhausted CD8+ T cells (CD8+ Tex) in NSCLC, CRC, HNSC, and LIHC, indicating that SEMA4D may suppress the immune effect of T cells.

### Relationship between SEMA4D expression and immune cell infiltration in diverse tumors

**Figure 3:**
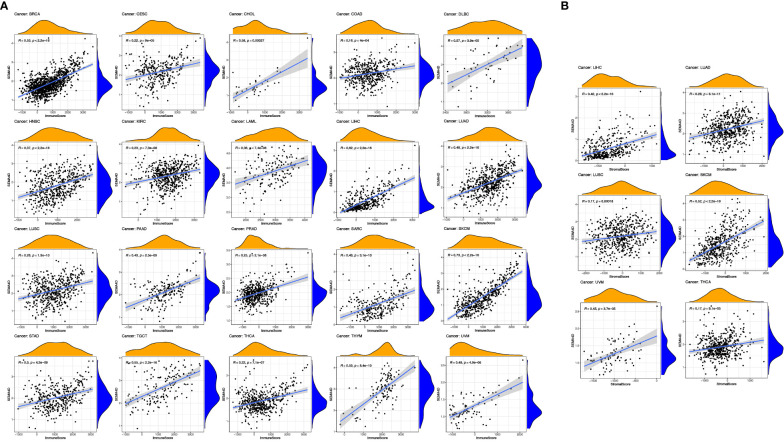
The high expression of *SEMA4D* was positively correlated with immune score and stromal scores. Correlation of *SEMA4D* expression with immune **(A)** and stromal scores **(B)** was displayed as above. *SEMA4D* expression showed positive correlation with immune cell infiltration in SKCM, HNSC, LAML, LUAD, PAAD, SARC, STAD, UVM, BRCA, CHOL, DLBC, LIHC, TGCT, THYM, CESC, COAD, KIRC, LUSC, PRAD and THCA. Meanwhile, in 6 tumor types, LIHC, LUAD, LUSC, SKCM, THYM and UVM, SEMA4D expression was positively correlated with stromal score.

The results of our study suggest a potential role of SEMA4D in immune function during tumor development. To explore this further, we analyzed the correlation between *SEMA4D* expression and the immune score and interstitial score in different tumors. As depicted in **Figure 3A**, *SEMA4D* expression was positively correlated with the immune score in 20 tumors. Notably, *SEMA4D* expression was strongly positively correlated with immune cell infiltration in SKCM and moderately positively correlated with immune cell infiltration in HNSC, acute myeloid leukaemia (LAML), LUAD, PAAD, sarcoma (SARC), stomach adenocarcinoma (STAD), uveal melanoma (UVM), breast invasive carcinoma (BRCA), cholangio carcinoma (CHOL), lymphoid neoplasm diffuse large B-cell lymphoma (DLBC), LIHC, TGCT, and thymoma (THYM). Additionally, cervical squamous cell carcinoma and endocervical adenocarcinoma (CESC), COAD, Kidney renal clear cell carcinoma (KIRC), Lung squamous cell carcinoma (LUSC), prostate adenocarcinoma (PRAD), and thyroid carcinoma (THCA) showed a weak positive correlation. These findings indicate that tumors with high *SEMA4D* expression are associated with a higher number of immune cells. Moreover, *SEMA4D* expression was positively correlated with stromal score in 6 tumor types, including LIHC, LUAD, LUSC, SKCM, THYM, and UVM, as shown in **Figure 3B**.

**Figure 4:**
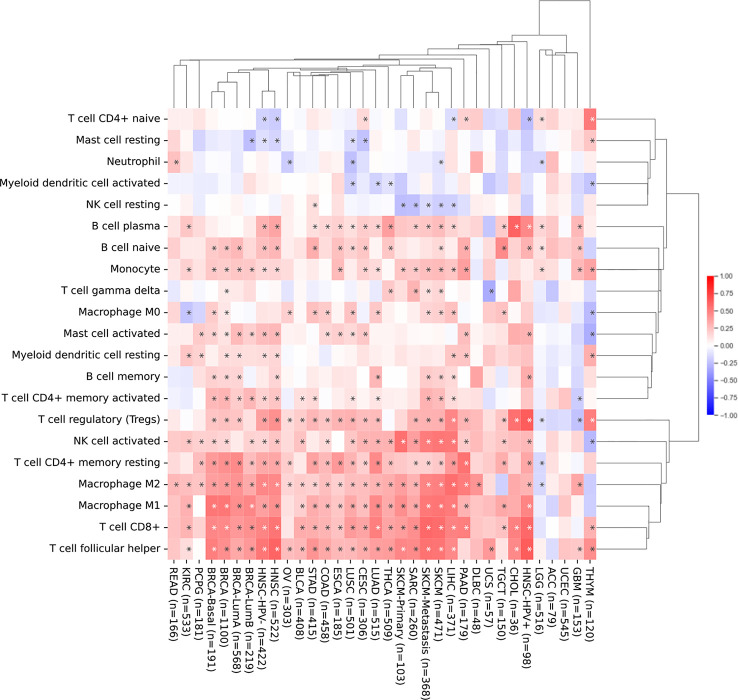
The infiltration of immune cell in the TME and SEMA4D expression are positively correlated in most cancers. Correlation analysis of *SEMA4D* expression and the infiltration of 21 immune cell types across different types of cancer from TCGA using CIBERSORT. The pattern shows the correlation between the number of immune cells and the expression of *SEMA4D* in TME. Red color means positive correlation between the number of immune cells and *SEME4D*, blue color means negative correlation between immune cells and *SEMA4D* expression, and the darker the color the stronger the correlation. The * in the diagram represents p value <0.05.

Next, we investigated the relationship between *SEMA4D* expression and infiltrating immune cells in the immune microenvironment using CIBERSORT, as shown in **Figure 4**. We found that *SEMA4D* was closely related to immune cell infiltration, and cluster analysis revealed three distinct clusters of immune cells, which were combined into two categories for practicality. Our analysis showed that a large fraction of immune cell types were highly enriched in cancer, including follicular helper T cells (26/34), CD8+ T cells (25/34), M1 macrophages (23/34), M2 macrophages (28/34), CD4+ memory resting T cells (24/34), activated NK cells (21/34), and regulatory T cells (24/34). Notably, one cluster of tumors showed a negative correlation between immune cell infiltration and *SEMA4D* expression, and these tumors were mostly found in the central nervous system or had low expression of *SEMA4D* itself. In summary, our findings indicate that *SEMA4D* expression is closely associated with the infiltration of multiple immune cell components in the tumor microenvironment.

### Correlation of SEMA4D expression with immune checkpoints, TMB and MSI

**Figure 5:**
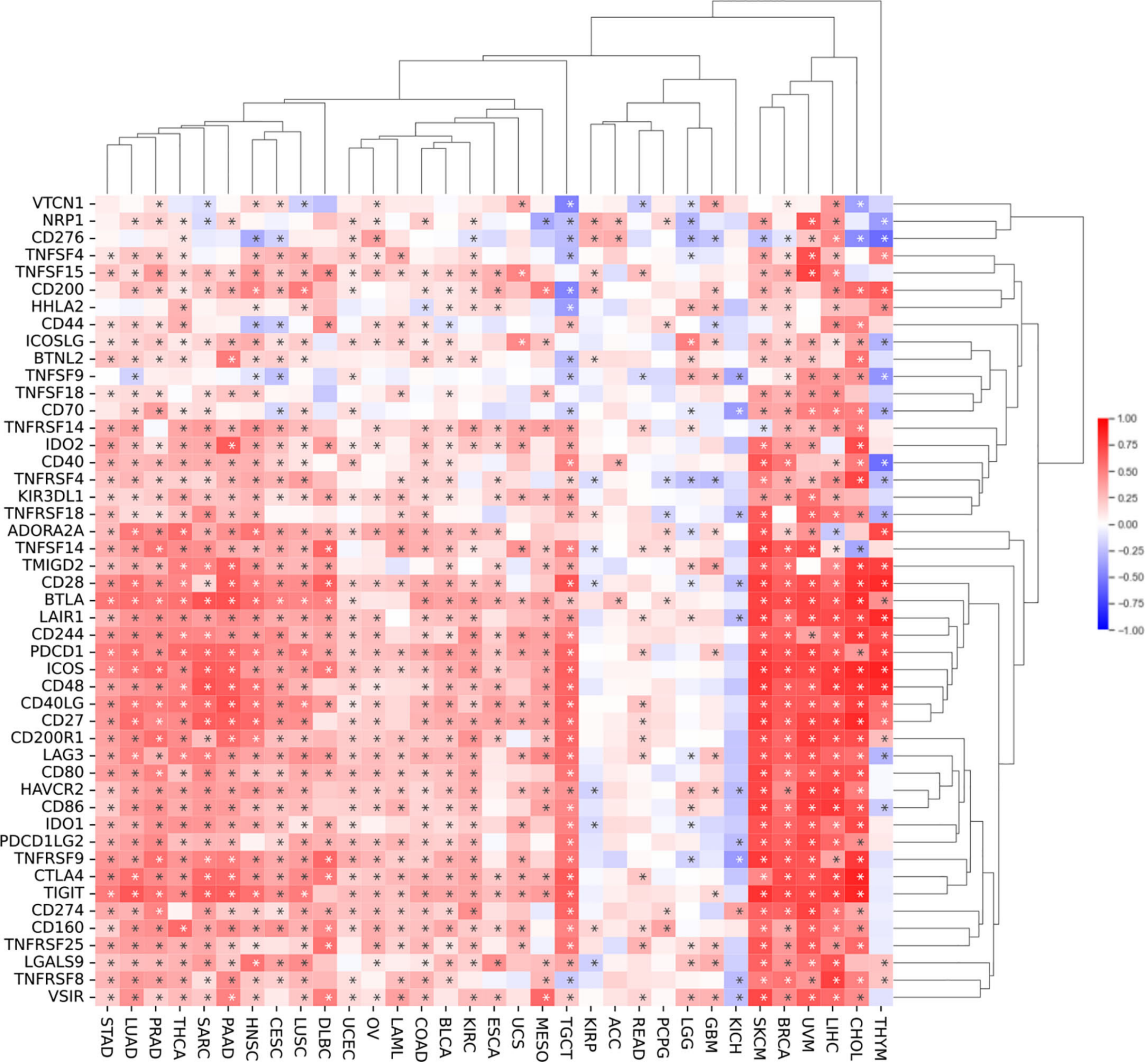
*SEMA4D* expression is broadly correlated with immune checkpoints. The pattern shows correlation analysis of *SEMA4D* expression and 47 immune checkpoint genes across cancers. *SEMA4D* was associated with more than 40 immune checkpoints in 13 tumors. Red color means positive correlation between checkpoints and *SEME4D*, blue color means negative correlation between checkpoints and *SEMA4D* expression, and the darker the color the stronger the correlation. The * in the diagram represents p value <0.05.

**Figure 6:**
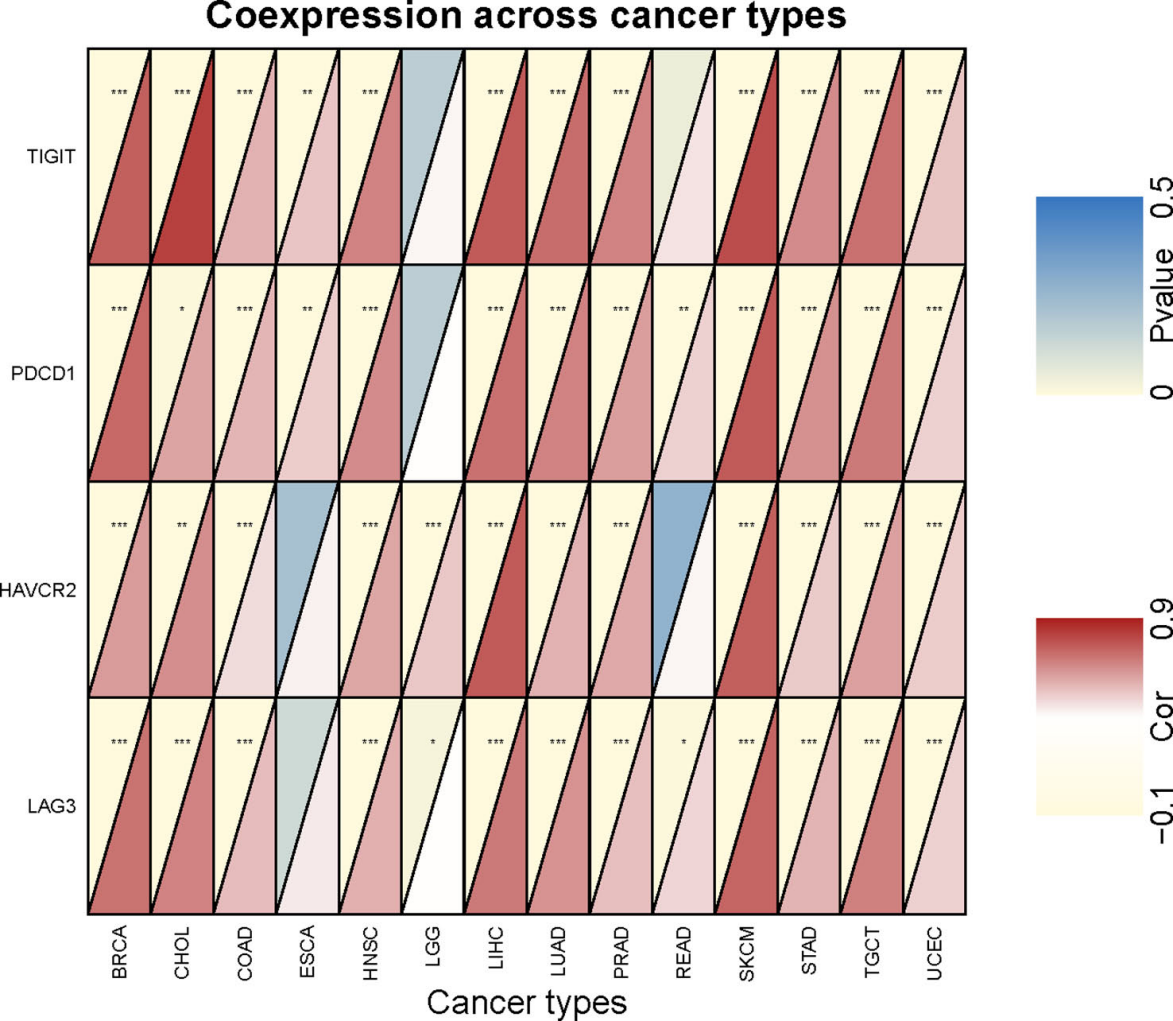
Correlation analysis of *SEMA4D* expression and CD8+ Tex signature checkpoint genes. The red depth in the lower right triangle of an individual rectangle represents the correlation between *SEMA4D* and the corresponding checkpoint in this type of tumor. *p < 0.05, **p < 0.01, and ***p < 0.001.

To investigate the relationship between *SEMA4D* expression and immune checkpoints, we analyzed the expression of 47 commonly used immune checkpoints in 20 different types of tumors (**Figure 5**). *SEMA4D* expression was broadly correlated with immune checkpoints, with more than 40 immune checkpoints showing a significant correlation with SEMA4D expression in the 13 tumors with the highest correlation. *SEMA4D* expression was significantly correlated with *PDCD1* (*PD1*) and *CD274* (*PDL1*) expression in most tumors. Additionally, *SEMA4D* expression exhibited a broad correlation with the expression of other immune checkpoints, including *CD28*, *BTLA*, *CD160*, *HAVCR2*, *LAG3*, *CTLA4*, *LAIR1*, *VSIR*, *CD200R*, *TIGIT*, *CD244*, *TNFRSF9*, *ADORA2A*, *ICOS*, *TNFSF14*, and *CD27*. The results suggest that blocking SEMA4D may enhance the efficacy of anti-PD1 and other immune checkpoint inhibitors. As *SEMA4D* was found to be associated with T cell exhaustion in the single-cell analysis shown in **Figure 2**, we further investigated the correlation between *SEMA4D* expression and CD8+ T cell exhaustion checkpoints (e.g., TIGIT, LAG3, TIM3, and PD1) in the 13 highly expressed *SEMA4D* tumor types (**Figure 6**). The results showed that except for brain lower grade glioma (LGG), *SEMA4D* expression was significantly positively correlated with CD8+ T cell exhaustion checkpoints.

**Figure 7:**
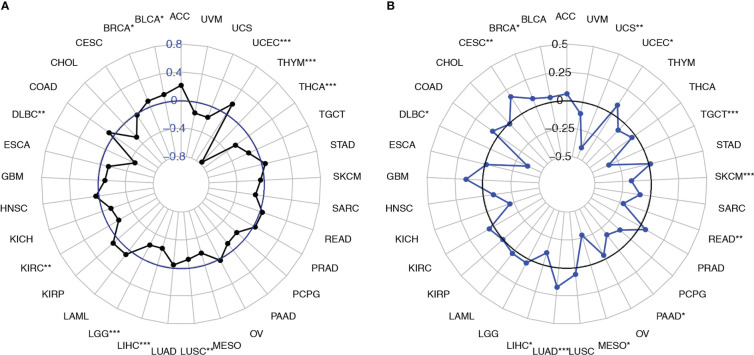
*SEMA4D* expression was correlated with the TMB and MSI. **(A)** Radar plots were constructed to show the correlation between *SEMA4D* expression and the TMB. The blue value represents the range, and the black curve represents the correlation coefficient. **(B)** Radar plots were constructed to show the correlation between *SEMA4D* expression and MSI. The black value represents the range, and the blue curve represents the correlation coefficient. *p < 0.05, **p < 0.01, and ***p < 0.001.

Next, we analyzed the correlation of *SEMA4D* expression with TMB and MSI. The results showed that *SEMA4D* expression was negatively correlated with TMB in most tumors, including DLBC, KIRC, LGG, LIHC, LUSC, THCA, and THYM, but was positively correlated with TMB in bladder urothelial carcinoma (BLCA), BRCA, and uterine corpus endometrial carcinoma (UCEC) (**Figure 7A**). *SEMA4D* expression was negatively correlated with MSI in most tumors, including LIHC, mesothelioma (MESO), PAAD, rectum adenocarcinoma (READ), SKCM, TGCT, and UCS, but was positively correlated with MSI in BRCA, CESC, LUAD, and UCEC (**Figure 7B**). These results suggest that the low TMB and MSI associated with low tumor neoantigen levels may be part of the immunosuppressive function exerted by SEMA4D.

### Validation of differential SEMA4D expression and distribution across cancers in TME

**Figure 8:**
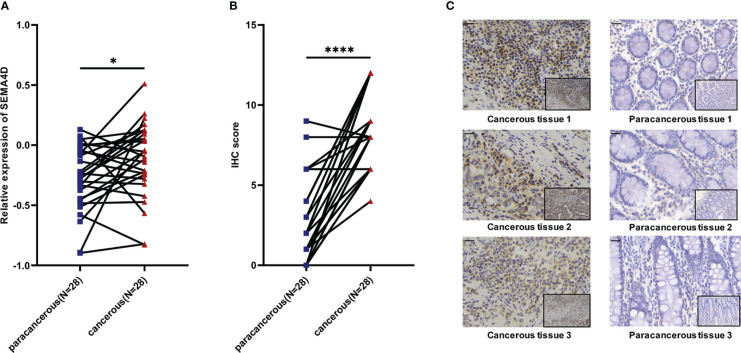
Expression of SEMA4D was higher in colorectal cancer than in normal colorectal tissue in clinical samples. **(A)** Relative expression of *SEMA4D* in 28 paired COAD cancerous tissues and paracancerous tissues determined using RT–qPCR. **(B)** IHC scores for SEMA4D in COAD tissues and paired normal tissues. **(C)** Representative images of IHC staining for SEMA4D in negative and positive samples were captured at 200x magnification on the bottom and 400x magnification on the top. Scale bar, 20 μm (top left corner). Data are shown as the means ± SD and were analyzed using Student’s t test and paired-samples t test. *p < 0.05, ****p < 0.0001.

We verified the expression of *SEMA4D* in tumors. We randomly selected 28 patients who were first diagnosed with colon cancer based on pathology in the Gastrointestinal Surgery Department of Shandong Provincial Hospital in September 2020 (excluding patients with chronic diseases or other malignant tumors and those who had received neoadjuvant chemoradiation). The cancer tissues and para carcinoma tissues from each patient were collected for IHC staining and RT–qPCR to explore the expression of the SEMA4D protein and mRNA. The results of RT–qPCR showed that the level of *SEMA4D* mRNA in tumor tissues was significantly higher than that in adjacent tissues (p=0.0269) (**Figure 8A**), and IHC staining proved that SEMA4D protein was also highly expressed in tumors (p<0.0001) (**Figures 8B, C**).

**Figure 9:**
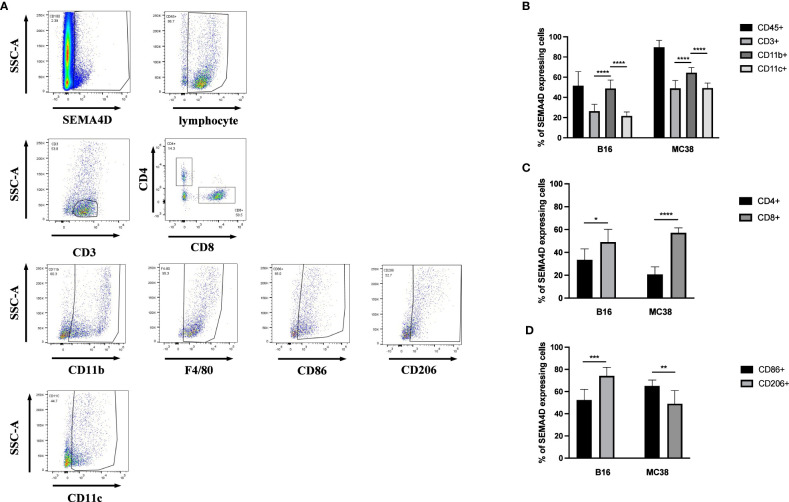
In MC38 and B16 subcutaneous transplantation tumor models in C57 mice, the primary source of SEMA4D was CD45+ cells. FCM analysis of SEMA4D expressing cells in MC38 tumors and B16 tumors isolated from C57BL/6 mice at 14 d postimplantation. **(A)** shows and describes the gating strategy for specimens and **(B–D)** is the result after quantifying. Bars show the means ± SD from n = 7–9 mice per group. *p < 0.05, **p < 0.01, ***p < 0.001, and ****p < 0.0001.

To explore the distribution of SEMA4D on immune cells, B16 and MC38 cells were implanted into female C57BL/6 mice (n=7-9) to specifically identify the cells expressing SEMA4D within the TME. Tumors were isolated for FCM analysis of SEMA4D+ cells after 14 days (**Figure 9A**). Consistent with the results from scRNA-seq datasets, SEMA4D expression was mainly observed in immunocytes. 89.62% ± 6.95% and 52.09% ± 12.91% immune cells showed SEMA4D staining in MC38 tumors and B16 tumors, respectively (**Figure 9B**).

We further analyzed the proportion of SEMA4D-positive cells in MC38 and B16 tumors, and the composition of SEMA4D+ cells in MC38 tumors was similar to that observed in B16 tumors. Among immune cells, myeloid cells (CD11B+) accounted for large proportions, including 49.47% ± 7.69% in B16 tumors (n=7) and 64.46% ± 5.10% in MC38 tumors (n=9) (**Figure 9B**). We also identified the cells expressing SEMA4D within several different categories of immune cells. The source of SEMA4D in CD3+ cells was predominantly CD8+ T cells in both B16 (47.47% ± 10.92%) and MC38 (57.19% ± 4.27%) tumors (**Figure 9C**). Among TAM (CD11B+ F4/80+), the distribution of SEMA4D was distinguished, with a greater proportion of M2 macrophages (CD206+) observed in B16 tumors (M1 = 52.37% ± 8.69%, M2 = 75.1% ± 7.36%), while a greater proportion of M1 macrophages (CD86+) was observed in MC38 tumors (M1 = 65.08% ± 5.31%, M2 = 49.02% ± 11.93%), and both differences were statistically significant (P<0.01) (**Figure 9D**).

### Inhibition of SEMA4D rescues T cell exhaustion

**Figure 10:**
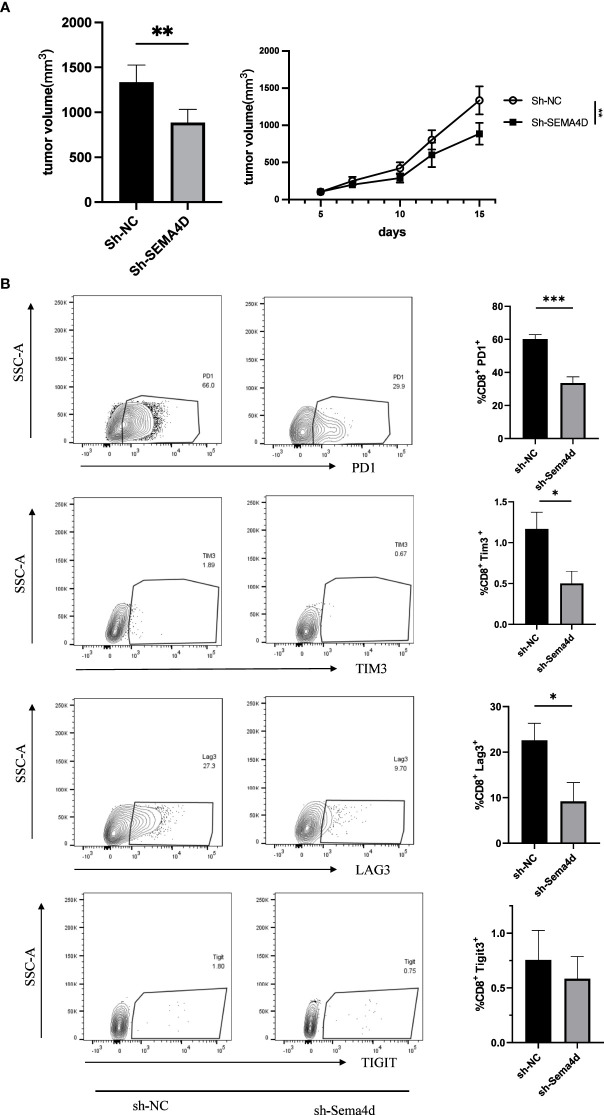
Reducing SEMA4D expression in tumors rescued CD8+ T cell exhaustion. **(A)** The growth rate and volume of in the B16sh-SEMA4D tumors were significantly reduced. **(B)** Expression of CD8+ Tex cell checkpoints was measured by FCM. PD1+, TIM3+ and LAG3+ T cells in B16sh-SEMA4D tumors were significantly decreased. Representative flow plots (left) and quantification (right) of the percentage of PD1+, TIM3+ and LAG3+ T cells in CD8+ T cells in the tumor. Data are presented as mean ± s.e.m (n = 5). *P<0.05; **P<0.01; ***P<0.001.

To investigate the functions of SEMA4D in the TME, we designed an *in vivo* experiment to test whether *SEMA4D* knockdown could rescue T cell exhaustion. We used lentiviral transfection to knock down *SEMA4D* in the B16 cell line and injected the cells subcutaneously into the back of C57 mice (n=5). Compared with the control group, we observed that tumor growth was significantly slower in the *SEMA4D* knockdown group (**Figure 10A**).

To further analyze the effects of *SEMA4D* knockdown on T cell exhaustion, we performed flow cytometry analysis of B16 tumors. We found that in *SEMA4D* knocked down tumors, PD1 expression in the TME decreased from 60.20 ± 2.61% to 33.60 ± 3.73% (P<0.05), LAG3 expression decreased from 22.58 ± 3.75% to 9.18 ± 4.14% (P<0.05), TIM3 expression decreased from 1.17 ± 0.20% to 0.50 ± 0.15% (P<0.05), and TIGIT expression decreased from 0.76 ± 0.27% to 0.58 ± 0.20% (P=0.62) (**Figure 10B**). These results indicate that reducing SEMA4D expression inhibits tumor growth by decreasing T cell exhaustion.

## Discussion

*SEMA4D* is an oncogene that functions in a variety of tumors, including colorectal carcinoma (14), cervical cancer (15), ovarian cancer (16), soft tissue carcinomas (17), oral cancer (18), esophageal squamous cell carcinoma (19), non-small cell lung cancer (2), osteosarcoma (20) and cutaneous squamous cell carcinoma (21), by binding to Plexin-B1, Plexin-B2 and CD72 receptors (22) and is associated with tumor growth and a poor prognosis. Our study verified these results, and the role of SEMA4D in TME was further investigated.

*SEMA4D* was not expressed at higher levels in all tumor types at advanced stages than in early-stage tumors. And *SEMA4D* was highly expressed in the early stage of LUAD, TGCT, KIRP, and THCA. This result seems to contradict the involvement of SEMA4D in tumor progression (15). These findings suggest that SEMA4D expression may not be directly correlated with tumor progression in all types of cancer. Further studies are needed to elucidate the precise role of SEMA4D in tumor progression in these specific cancer types. However, tumor tissue has a complex immune microenvironment. The TME, comprising interactions between proliferating neoplastic cells and stromal components, is critical for tumor growth according to the seed and soil theory proposed by Stephen Paget (23). Our findings suggest that *SEMA4D* is associated with the infiltration of immune cells, *SEMA4D* expression was positively correlated with the immune score in 20 tumors, and positively correlated with the stromal score in 6 types of tumors. Then, *SEMA4D* is mainly produced by immune cells in the TME. We believed that the downregulation of *SEMA4D* expression as the tumor progresses might result from decreased immune cell infiltration.

SEMA4D has been shown to be widely expressed on immune cells (22), but its specific distribution in the immune microenvironment has not been systematically analyzed. The spatial distribution of immune cells in the tumor also directly affects the antitumor immune response. Our previous study described the presence of gradient changes in SEMA4D expression in infiltrating immune cells at the tumor edge in human colorectal cancer (24). The expression of SEMA4D at the edge of invasive tumors creates a barrier for immune cell infiltration and alters the balance between regulatory and effector immune cells and signals (3).

We used single-cell RNA-seq transcriptome data to show that *SEMA4D* is widely expressed in multiple immune cell components, especially macrophages. We subsequently found similar results in two implanted tumor cells with a significant proportion of CD11B+ myeloid cells and CD11C+ DCs among SEMA4D+ CD45+ cells. However, significant differences were observed in the TAM between the two tumors. SEMA4D expression levels were predominant on M2 macrophages in B16 tumors, whereas M1 SEMA4D+ cells were more abundant in MC38 tumors.

In addition, we noted that *SEMA4D* was widely expressed in CD8+ exhausted T cells in single-cell sequencing data. And *SEMA4D* showed substantial correlation with multiple immune checkpoints in a dozen tumors, including *PDCD1* (*PD1*), *CD274* (*PDL1*), *CD28*, *BTLA*, *HAVCR2* (*TIM3*), *LAG3*, *CTLA4*, *TIGIT*, *ICOS*, *CD27* and other common immune checkpoints. Among them, *PDCD1* (*PD1*), *CD274* (*PDL1*), *HAVCR2* (*TIM3*), *LAG3*, and *TIGIT* are associated with T-cell exhaustion (25). Usually, exhaustion of tumor-specific CD8+ T cells leads to impairment of cytokine secretion and proliferation capacity, accompanied by PD1, TIM3, LAG3 and TIGIT overexpression (26). And the severity of T-cell exhaustion is influenced by the number of inhibitory receptors co-expressed by exhausted T cells (27). Furthermore, considering that since SEMA4D is expressed in LGG myelin and oligodendrocytes (25), it still shows some correlation with TIM3 expression. Our results showed that *SEMA4D* knockdown delays tumor growth and resulted in a significant decrease in PD1, LAG3, and TIM3 expression in TME, and a trend of decreased TIGIT expression. Thus, the relationship between SEMA4D and T cell exhaustion in TME was confirmed. We suggest that the reduction of SEMA4D expression in tumors can rescue CD8+T cells exhaustion in the TME. Rafael et al. previously reported that SEMA4D was associated with T-cell exhaustion during HIV-1 infection (28), which coincides with our results. In the process of tumor development, SEMA4D causes T cell exhaustion, leading to a suppressive immune microenvironment with reduced immune cell infiltration, thereby promoting tumor growth. Our results also suggest that decreased *SEMA4D* expression after tumor progression to advanced stages may be associated with decreased immune cell infiltration.

SEMA4D expressed on tumor cells very likely affects immune cells. Younis reported that SEMA4D secreted by human HNSC cells substantially inhibits T cell proliferation and function (29). SEMA4D is expressed at high levels on MOC1 oral cancer cells. Blocking MOC1 oral cancer cells SEMA4D does not directly affect tumor cell proliferation but reduces the production of chemokines by tumor cells, resulting in reduced MDSC and enhanced T lymphocyte response (18). In our experiments, SEMA4D expressed on tumor cells plays an important role in inducing an inhibitory immune effects. Thus, the immune reprogramming process induced by SEMA4D signaling between tumor cells and immune cells plays an important role in tumor immunity.

The TMB and MSI are important biomarkers for predicting the response of tumors to immunotherapy. Our results indicated that *SEMA4D* mostly negatively correlated with TMB and MSI in tumors was. Thus, instead of interfering with the immune response by affecting tumor neoantigenesis, *SEMA4D* may lead to immunosuppression mainly by interfering with immune checkpoints in the microenvironment, affecting the effector function of T cells against tumor cells.

Therefore, blocking SEMA4D may be able to improve the efficacy of immune antitumor therapy. Pepinemab (an anti-SEMA4D antibody) has achieved considerable antitumor therapeutic effects on resectable pancreatic and colon cancer (30). Anti-SEMA4D antibody combined with immunomodulators such as CTLA4 or PD1/PDL1 monoclonal antibodies enhanced the trafficking of killer macrophages and activated CD8+ T cells to the TME, enhanced the efficacy of immune checkpoint blockers (3, 31). The effectiveness of immune checkpoint inhibitor (ICI) therapy is critically dependent on the expansion of T cells and the subsequent increase in infiltration of precursor-exhausted CD8+ T cells within the tumor (32), and there is potential to further reduce T cell exhaustion by targeting SEMA4D and thereby synergistically promote the effectiveness of ICI therapy. In addition, the evidence of *SEMA4D* expression in various immune cell subsets from single-cell datasets is still insufficient. It is necessary to further clarify the expression of *SEMA4D* and its inhibitory function in immune cell subsets within the tumor microenvironment using single-cell sequencing technology in order to identify the targets and mechanisms of anti-SEMA4D immunotherapy, and ultimately achieve the goal of promoting the therapeutic effect of combination immunotherapy with ICI.

In conclusion, we explored SEMA4D expression patterns with bioinformatics methods and determined its relationship with immune cell infiltration and with CD8+Tex cells, providing a new perspective for exploring the role of SEMA4D in tumor immunity. However, detection in large tumor tissues limits our analysis of the temporal and spatial distribution of SEMA4D on immune cells and tumor cells. In the future, single-cell sequencing and spatial transcriptome sequencing technologies are needed for further analysis (33).

## Data availability statement

The original contributions presented in the study are included in the article/**Supplementary Materials**, further inquiries can be directed to the corresponding authors.

## Ethics statement

The studies involving human participants were reviewed and approved by Medical Ethics Committee of Shandong Provincial Hospital. The patients/participants provided their written informed consent to participate in this study. The animal study was reviewed and approved by Committee for Ethics of Animal Experiments of Shandong Provincial Hospital.

## Author contributions

SZ designed the study and wrote the manuscript. ZZ and CY collected clinical data. JW revised the manuscript and had final approval of the submitted and published versions. CJ and LL were guarantors of this work and, as such, had full access to all the data in the study and take responsibility for the integrity of the data and the accuracy of the data analysis. All authors covntributed to the article and approved the submitted version.
