# Supplementary material for: Mediating immunosuppressive functions: a new perspective on the complex immunological properties of SEMA4D in the tumor microenvironment
Source: Front Oncol. 2023 May 23;13:1171926. doi: 10.3389/fonc.2023.1171926 (PMC10242174; doi:10.3389/fonc.2023.1171926)
Supplement: Supplementary file 1 [file DataSheet_1.docx]

The datasets used in our analysis are publicly available for download at [UCSC Xena (xenabrowser.net)](https://xenabrowser.net/datapages/). Specifically, the datasets we used were as follows:

[GDC TCGA Acute Myeloid Leukemia (LAML) (xenabrowser.net)](https://xenabrowser.net/datapages/?cohort=GDC%20TCGA%20Acute%20Myeloid%20Leukemia%20(LAML)&removeHub=https%3A%2F%2Fxena.treehouse.gi.ucsc.edu%3A443)

[GDC TCGA Adrenocortical Cancer (ACC) (xenabrowser.net)](https://xenabrowser.net/datapages/?cohort=GDC%20TCGA%20Adrenocortical%20Cancer%20(ACC)&removeHub=https%3A%2F%2Fxena.treehouse.gi.ucsc.edu%3A443)

[GDC TCGA Bile Duct Cancer (CHOL) (xenabrowser.net)](https://xenabrowser.net/datapages/?cohort=GDC%20TCGA%20Bile%20Duct%20Cancer%20(CHOL)&removeHub=https%3A%2F%2Fxena.treehouse.gi.ucsc.edu%3A443)

[GDC TCGA Bladder Cancer (BLCA) (xenabrowser.net)](https://xenabrowser.net/datapages/?cohort=GDC%20TCGA%20Bladder%20Cancer%20(BLCA)&removeHub=https%3A%2F%2Fxena.treehouse.gi.ucsc.edu%3A443)

[GDC TCGA Breast Cancer (BRCA) (xenabrowser.net)](https://xenabrowser.net/datapages/?cohort=GDC%20TCGA%20Breast%20Cancer%20(BRCA)&removeHub=https%3A%2F%2Fxena.treehouse.gi.ucsc.edu%3A443)

[GDC TCGA Cervical Cancer (CESC) (xenabrowser.net)](https://xenabrowser.net/datapages/?cohort=GDC%20TCGA%20Cervical%20Cancer%20(CESC)&removeHub=https%3A%2F%2Fxena.treehouse.gi.ucsc.edu%3A443)

[GDC TCGA Colon Cancer (COAD) (xenabrowser.net)](https://xenabrowser.net/datapages/?cohort=GDC%20TCGA%20Colon%20Cancer%20(COAD)&removeHub=https%3A%2F%2Fxena.treehouse.gi.ucsc.edu%3A443)

[GDC TCGA Endometrioid Cancer (UCEC) (xenabrowser.net)](https://xenabrowser.net/datapages/?cohort=GDC%20TCGA%20Endometrioid%20Cancer%20(UCEC)&removeHub=https%3A%2F%2Fxena.treehouse.gi.ucsc.edu%3A443)

[GDC TCGA Esophageal Cancer (ESCA) (xenabrowser.net)](https://xenabrowser.net/datapages/?cohort=GDC%20TCGA%20Esophageal%20Cancer%20(ESCA)&removeHub=https%3A%2F%2Fxena.treehouse.gi.ucsc.edu%3A443)

[GDC TCGA Glioblastoma (GBM) (xenabrowser.net)](https://xenabrowser.net/datapages/?cohort=GDC%20TCGA%20Glioblastoma%20(GBM)&removeHub=https%3A%2F%2Fxena.treehouse.gi.ucsc.edu%3A443)

[GDC TCGA Head and Neck Cancer (HNSC) (xenabrowser.net)](https://xenabrowser.net/datapages/?cohort=GDC%20TCGA%20Head%20and%20Neck%20Cancer%20(HNSC)&removeHub=https%3A%2F%2Fxena.treehouse.gi.ucsc.edu%3A443)

[GDC TCGA Kidney Chromophobe (KICH) (xenabrowser.net)](https://xenabrowser.net/datapages/?cohort=GDC%20TCGA%20Kidney%20Chromophobe%20(KICH)&removeHub=https%3A%2F%2Fxena.treehouse.gi.ucsc.edu%3A443)

[GDC TCGA Kidney Clear Cell Carcinoma (KIRC) (xenabrowser.net)](https://xenabrowser.net/datapages/?cohort=GDC%20TCGA%20Kidney%20Clear%20Cell%20Carcinoma%20(KIRC)&removeHub=https%3A%2F%2Fxena.treehouse.gi.ucsc.edu%3A443)

[GDC TCGA Kidney Papillary Cell Carcinoma (KIRP) (xenabrowser.net)](https://xenabrowser.net/datapages/?cohort=GDC%20TCGA%20Kidney%20Papillary%20Cell%20Carcinoma%20(KIRP)&removeHub=https%3A%2F%2Fxena.treehouse.gi.ucsc.edu%3A443)

[GDC TCGA Large B-cell Lymphoma (DLBC) (xenabrowser.net)](https://xenabrowser.net/datapages/?cohort=GDC%20TCGA%20Large%20B-cell%20Lymphoma%20(DLBC)&removeHub=https%3A%2F%2Fxena.treehouse.gi.ucsc.edu%3A443)

[GDC TCGA Liver Cancer (LIHC) (xenabrowser.net)](https://xenabrowser.net/datapages/?cohort=GDC%20TCGA%20Liver%20Cancer%20(LIHC)&removeHub=https%3A%2F%2Fxena.treehouse.gi.ucsc.edu%3A443)

[GDC TCGA Lower Grade Glioma (LGG) (xenabrowser.net)](https://xenabrowser.net/datapages/?cohort=GDC%20TCGA%20Lower%20Grade%20Glioma%20(LGG)&removeHub=https%3A%2F%2Fxena.treehouse.gi.ucsc.edu%3A443)

[GDC TCGA Lung Adenocarcinoma (LUAD) (xenabrowser.net)](https://xenabrowser.net/datapages/?cohort=GDC%20TCGA%20Lung%20Adenocarcinoma%20(LUAD)&removeHub=https%3A%2F%2Fxena.treehouse.gi.ucsc.edu%3A443)

[GDC TCGA Lung Squamous Cell Carcinoma (LUSC) (xenabrowser.net)](https://xenabrowser.net/datapages/?cohort=GDC%20TCGA%20Lung%20Squamous%20Cell%20Carcinoma%20(LUSC)&removeHub=https%3A%2F%2Fxena.treehouse.gi.ucsc.edu%3A443)

[GDC TCGA Melanoma (SKCM) (xenabrowser.net)](https://xenabrowser.net/datapages/?cohort=GDC%20TCGA%20Melanoma%20(SKCM)&removeHub=https%3A%2F%2Fxena.treehouse.gi.ucsc.edu%3A443)

[GDC TCGA Mesothelioma (MESO) (xenabrowser.net)](https://xenabrowser.net/datapages/?cohort=GDC%20TCGA%20Mesothelioma%20(MESO)&removeHub=https%3A%2F%2Fxena.treehouse.gi.ucsc.edu%3A443)

[GDC TCGA Ocular melanomas (UVM) (xenabrowser.net)](https://xenabrowser.net/datapages/?cohort=GDC%20TCGA%20Ocular%20melanomas%20(UVM)&removeHub=https%3A%2F%2Fxena.treehouse.gi.ucsc.edu%3A443)

[GDC TCGA Ovarian Cancer (OV) (xenabrowser.net)](https://xenabrowser.net/datapages/?cohort=GDC%20TCGA%20Ovarian%20Cancer%20(OV)&removeHub=https%3A%2F%2Fxena.treehouse.gi.ucsc.edu%3A443)

[GDC TCGA Pancreatic Cancer (PAAD) (xenabrowser.net)](https://xenabrowser.net/datapages/?cohort=GDC%20TCGA%20Pancreatic%20Cancer%20(PAAD)&removeHub=https%3A%2F%2Fxena.treehouse.gi.ucsc.edu%3A443)

[GDC TCGA Rectal Cancer (READ) (xenabrowser.net)](https://xenabrowser.net/datapages/?cohort=GDC%20TCGA%20Rectal%20Cancer%20(READ)&removeHub=https%3A%2F%2Fxena.treehouse.gi.ucsc.edu%3A443)

[GDC TCGA Sarcoma (SARC) (xenabrowser.net)](https://xenabrowser.net/datapages/?cohort=GDC%20TCGA%20Sarcoma%20(SARC)&removeHub=https%3A%2F%2Fxena.treehouse.gi.ucsc.edu%3A443)

[GDC TCGA Stomach Cancer (STAD) (xenabrowser.net)](https://xenabrowser.net/datapages/?cohort=GDC%20TCGA%20Stomach%20Cancer%20(STAD)&removeHub=https%3A%2F%2Fxena.treehouse.gi.ucsc.edu%3A443)

[GDC TCGA Testicular Cancer (TGCT) (xenabrowser.net)](https://xenabrowser.net/datapages/?cohort=GDC%20TCGA%20Testicular%20Cancer%20(TGCT)&removeHub=https%3A%2F%2Fxena.treehouse.gi.ucsc.edu%3A443)

[GDC TCGA Thymoma (THYM) (xenabrowser.net)](https://xenabrowser.net/datapages/?cohort=GDC%20TCGA%20Thymoma%20(THYM)&removeHub=https%3A%2F%2Fxena.treehouse.gi.ucsc.edu%3A443)

[GDC TCGA Thyroid Cancer (THCA) (xenabrowser.net)](https://xenabrowser.net/datapages/?cohort=GDC%20TCGA%20Thyroid%20Cancer%20(THCA)&removeHub=https%3A%2F%2Fxena.treehouse.gi.ucsc.edu%3A443)

[GDC TCGA Uterine Carcinosarcoma (UCS) (xenabrowser.net)](https://xenabrowser.net/datapages/?cohort=GDC%20TCGA%20Uterine%20Carcinosarcoma%20(UCS)&removeHub=https%3A%2F%2Fxena.treehouse.gi.ucsc.edu%3A443)

[GDC TCGA Prostate Cancer (PRAD) (xenabrowser.net)](https://xenabrowser.net/datapages/?cohort=GDC%20TCGA%20Prostate%20Cancer%20(PRAD)&removeHub=https%3A%2F%2Fxena.treehouse.gi.ucsc.edu%3A443)

[GDC TCGA Pheochromocytoma & Paraganglioma (PCPG) (xenabrowser.net)](https://xenabrowser.net/datapages/?cohort=GDC%20TCGA%20Pheochromocytoma%20%26%20Paraganglioma%20(PCPG)&removeHub=https%3A%2F%2Fxena.treehouse.gi.ucsc.edu%3A443)
