# Supplementary figures and images for: Mediating immunosuppressive functions: a new perspective on the complex immunological properties of SEMA4D in the tumor microenvironment
Source: Front Oncol. 2023 May 23;13:1171926. doi: 10.3389/fonc.2023.1171926 (PMC10242174; doi:10.3389/fonc.2023.1171926)

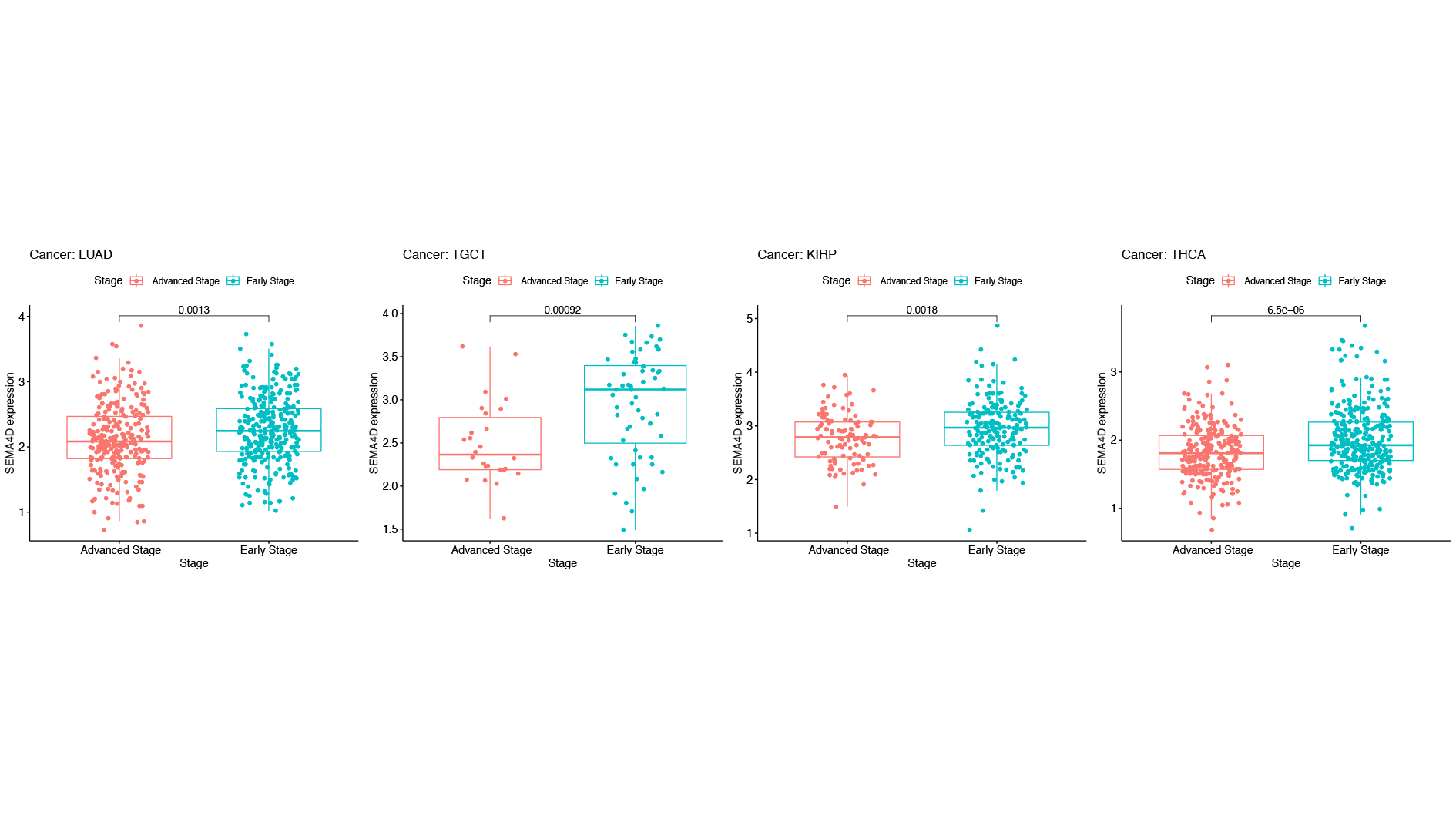

Supplement: Supplementary file 2 [file Image_1.tif]

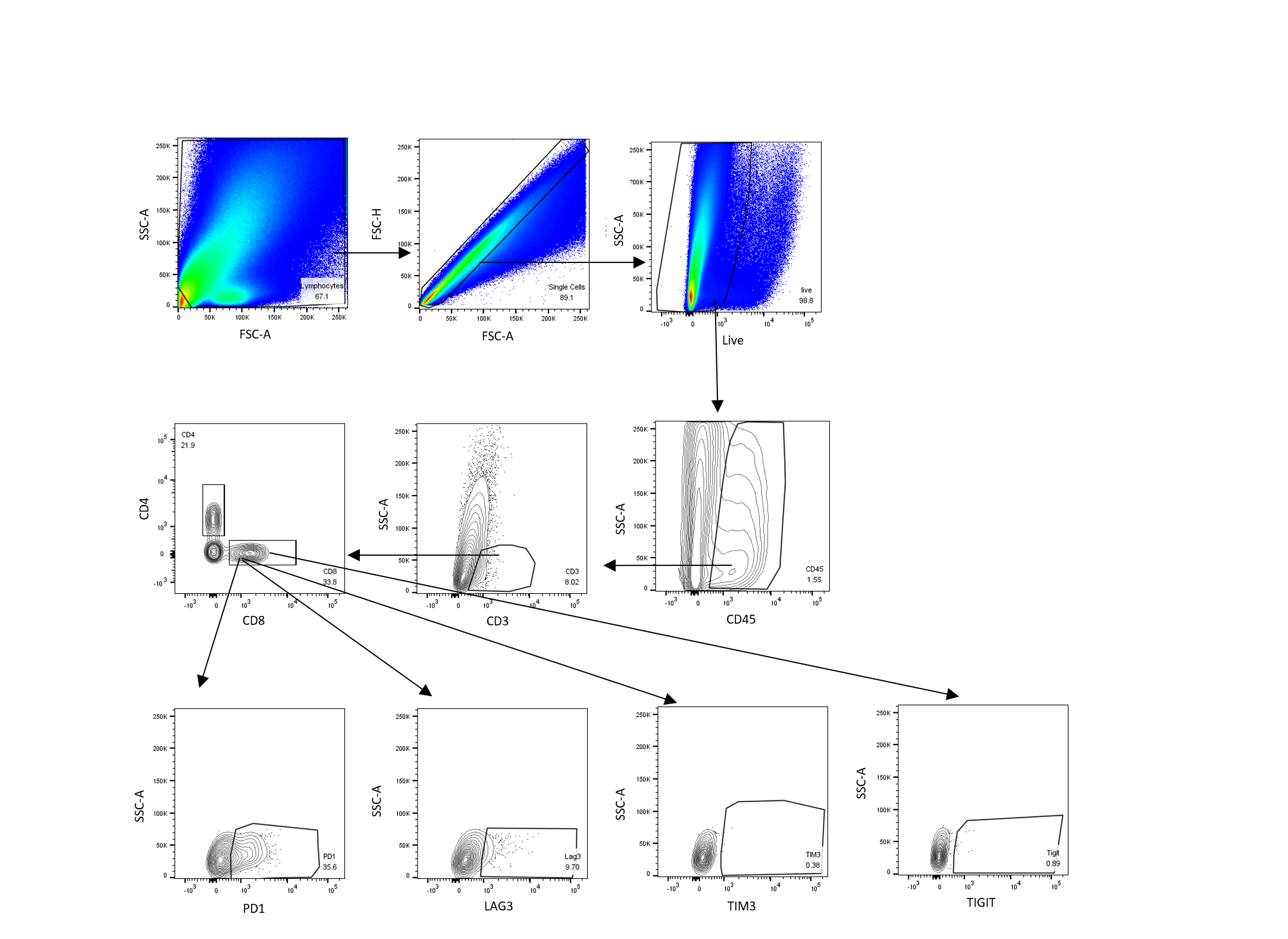

Supplement: Supplementary file 3 [file Image_2.tif]

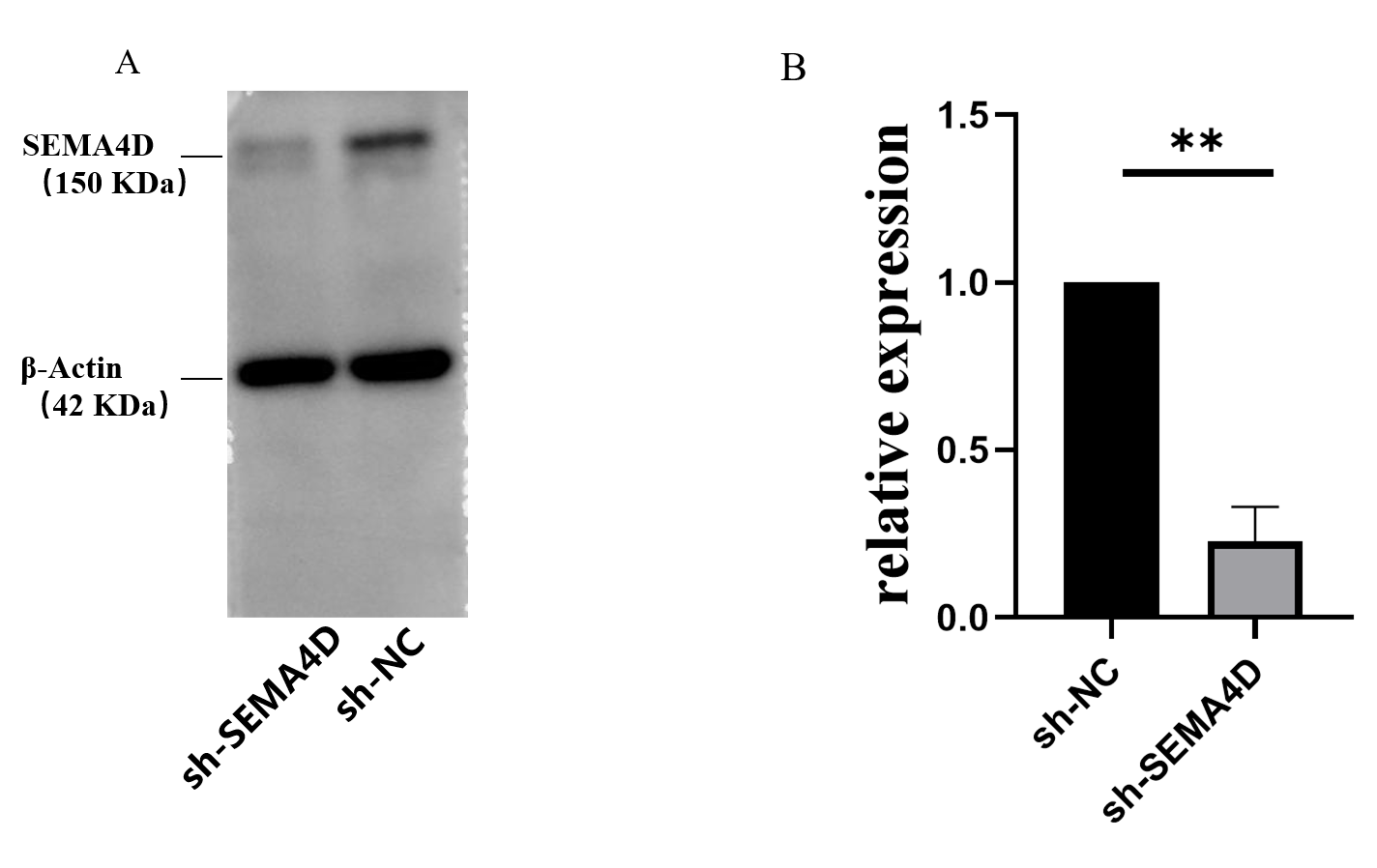

Supplement: Supplementary file 4 [file Image_3.tif]
