# Supplementary material for: Mediating immunosuppressive functions: a new perspective on the complex immunological properties of SEMA4D in the tumor microenvironment
Source: Front Oncol. 2023 May 23;13:1171926. doi: 10.3389/fonc.2023.1171926 (PMC10242174; doi:10.3389/fonc.2023.1171926)
Supplement: Supplementary file 5 [file Table_1.docx]

# Supplementary table1

Knockdown mice SEMA4D lentivirus interfering sequence is GCTGATGAGTGAGGACAAAGA. Antibody of WB for verified the knockdown efficiency was purchased from CST (53108 & 3700). The qRT-PCR primer sequences were as follows:

| RT–qPCR primer sequences for evaluate B16 knockdown efficiency | M_GAPDH-Forward | TGAAGGTCGGTGTGAACGGATT |
| --- | --- | --- |
|  | M_GAPDH-Reverse | CGTGAGTGGAGTCATACTGGAACA |
|  | M_SEMA4D-Forward | CACGGTGGAGGCAGTCTTCT |
|  | M_SEMA4D-Reverse | GGGAGATTCAAGGAGCTGGTG |
